# A facile chemical synthesis of Cu_x_Ni_(1−x)_Fe_2_O_4_ nanoparticles as a nonprecious ferrite material for electrocatalytic oxidation of acetaldehyde

**DOI:** 10.1038/s41598-020-59655-3

**Published:** 2020-02-17

**Authors:** Mai M. Khalaf, Hany M. Abd El-Lateef, Ahmed O. Alnajjar, Ibrahim M. A. Mohamed

**Affiliations:** 1 Department of Chemistry, College of Science, King Faisal University, P.O. Box 380 Al Hofuf, 31982 Al-Ahsa, Saudi Arabia; 2 Department of Chemistry, Faculty of Science, Sohag university, Sohag, 82524 Egypt

**Keywords:** Chemistry, Energy science and technology, Materials science

## Abstract

In the present work, Cu-doped nickel ferrite (Cu_x_Ni_(1−x)_Fe_2_O_4_) nanoparticles (CuNFNPs) were chemically fabricated by adding citric acid as a capping agent followed by combustion and calcination for acetaldehyde oxidation reaction (AOR) in KOH electrolytes. The as-prepared CuNFNPs were studied in terms of Fourier-transform infrared spectroscopy (FT-IR), Transmission electron microscopy (TEM), Field emission scanning electron microscope (FE-SEM), Energy-dispersive X-ray spectroscopy (EDX), X-ray diffraction (XRD) and Brunauer-Emmett-Teller (BET) specific surface area analyses. The morphology of CuNFNPs has sponges-structure containing irregular pores. Additionally, XRD analysis indicated that the prepared CuNFNPs have a cubic-crystals ferrite without the existence of impurities and the crystal size around 20.2 nm. The electrooxidation of acetaldehyde by the presented CuNFNPs was investigated using cyclic voltammetry (CV), chronoamperometry (CA) and electrochemical impedance spectroscopy (EIS) in ^−^OH media. Furthermore, the effects of ^−^OH and acetaldehyde on the electrocatalytic performance were studied with and without Cu-doping in addition to EIS and CA studies which confirm the high-performance of CuNFNPs as an electrocatalyst for AOR.

## Introduction

Electrocatalytic degradation of aldehydes which have the highest potential among the volatile organic compounds continues to be one of the interesting research points because it can be utilized for different catalytic industrial application areas^1^. Aldehydes including acetaldehyde can be produced through alcohol electrooxidation besides the degradation of contaminated organic compounds^2^. It’s a combustion intermediate of biofuels and fossil fuels and biofuels^3^. Due to aldehydes emission ability to the outer atmosphere, it is important to remove aldehydes before its emission. Electrocatalytic oxidation can be applied and considered as one of the best techniques because of low-operating temperature, and energy consumption. In spite of the reported literature, the research on the acetaldehyde electrooxidation remains far from commercialization. Compared to ethanol, methanol, formic acid, and urea, the acetaldehyde electrooxidation reaction (AOR) was scarcely reported in the literature^4,5^. Therefore, this study focusses on the AOR at the surface of novel and non-precious material.

Acetaldehyde (CH_3_–CH=O) is the simplest structure having C–C-species with different polarity and, as such, may be easily studied as a model for the investigation of C-C bond cleavage. Therefore, it would be worth studying how the aldehydes compounds which may produce as intermediates in fuel cells could be oxidized to CO_2_. The AOR has similar intermediates of ethanol (CH_3_–CH_2_OH) electrooxidation which is the anodic process of ethanol fuel cells (EFCs), i.e. CH_3_CO_(ads)_, CH_x(ads)_ and CO_(ads)_^4,6^. These intermediates can finally produce acetic acid and/or CO_2_. Therefore, the investigation of AOR appears to have a high potential in the process of EFCs. The utilized electrocatalyst for AOR mostly to be precious metals including Pt or Pt-alloys which can play an urgent role for commercialization of AOR^7,8^. Thus, finding non-precious catalysts for AOR can facilitate the industrial applications of AOR. In this work, non-precious ferrite material was investigated for an efficient AOR.

Nanoparticles of non-precious ferrites (MFe_2_O_4_) have promising physical characteristics such as electrical and magnetic properties and have a high potential to be utilized in many recent applications. However, the properties mainly depend upon the combined divalent metal with Fe as well as the size of the prepared particles. Among the divalent metals of ferrites, Ni-ferrite (NiFe_2_O_4_) has much interest because it’s a distinctive electrocatalyst, soft semiconductor, ferromagnetic, has high conductivity and stability^9^. Its composition has an inverse spinel structure, in which the Ni^2+^ ions are distributed in the octahedral corners, while ferric-ions are located at both octahedral and tetrahedral places^10^. Its chemical design was reported by many methods which show variable characteristics and performances^11^. The NiFe_2_O_4_ particles were introduced for many applications such as gas sensor^12^, Li-ion storage^13^, preparation of biphenyls^14^, acetone sensor^15^, sorbent for oils/organic pollutants^16^, photocatalytic degradation^17^, water splitting^18^, and in electrocatalysis for hydrogen evolution^19^, hydrazine oxidation^20^ and oxygen evolution^21^. Herein, this work tries to present a simple methodology to design non-precious or Pt-free NiFe_2_O_4_ nanoparticles and then, investigated for electrocatalysis of AOR. The sol-gel methodology was utilized to prepare the presented material as well as introducing Cu-doping. Doping of Cu in the NiFe_2_O_4_ material was studied in terms of physical, chemical and AOR electrocatalytic performance. The AOR was investigated by the simple ferrite in addition to Cu-doped Ni-ferrite at the glassy carbon electrode in high pH-media.

## Experimental

### Chemicals and solutions

Iron nitrate (Fe(NO_3_)_3_.9H_2_O), Copper (II) nitrate, Cu(NO_3_)_2_, anhydrous citric acid (C_6_H_8_O_7_), nickel nitrate (Ni(NO_3_)_2_.6H_2_O), acetaldehyde (CH_3_CHO), and 30% ammonia solution were found through Sigma Aldrich Co. Ltd., USA. All acetaldehyde/KOH solutions were prepared by the use of bidistilled water as a solvent and the estimated amount of chemicals. A commercial glassy carbon electrode (GCE) with a diameter equal to 3 mm and area around 0.071 cm^2^ was obtained from CH Instruments, Inc., USA. All chemicals utilized without any further refining.

### Chemical fabrication of ferrite material

Cu-Ni-ferrite according to the composition (Cu_x_Ni_(1−x)_Fe_2_O_4_; CuNFNPs) (x = 0.2) were synthesized by the sol-gel auto-combustion method. The experimental steps started by preparing solutions of iron nitrate (0.01 M) and nickel nitrate (0.01 M) by dissolving a calculated amount of iron nitrate in 50 ml of distilled water and also from nickel nitrate in 50 ml of distilled water. Both solutions were stirred very well to get homogeneous solutions. Under the stirring of iron nitrate solution, a nickel nitrate solution was added. Then, a stoichiometric ratio of copper nitrate was added, which was followed by adding a measured amount of citric acid as a capping agent dissolved in 5 ml of distilled water. The measured weight of citric acid corresponds to the molar ratio of 1.5:1. After that, stirring lasted for an additional 15 minutes before adding the ammonia to adjust the pH value to be in the range of 8 to 9. Then, the stirring continued for an additional one hour besides heating the solution at 70 °C. The obtained solution was covered and aged for 12 hours. This was the first stage of the synthesis steps which was the gel formation stage. The next stage was the thermal treatment of the obtained gel which was started by drying the gel at 100 °C for 10 hours in an oven. Then, the combustion of the obtained deposit was done at 180 °C for an hour. The obtained black fragile mass material was grinded to the powder. The last stage is the calcination process. The grinded powder was calcined at 400 °C for three hours. The obtained CuNFNPs material was studied for physical, chemical and performance investigation as an electrocatalyst for acetaldehyde oxidation.

### Characterization of the prepared catalyst

The morphology was checked via a field emission scanning electron microscope (FE-SEM) JSM-5410 and a transmission electron microscope (TEM) and (Model-JEOL, Japan). The chemical specification was analyzed via EDX (energy dispersive X-ray analysis). The crystallinity of CuNFNPs was investigated by X-ray diffraction (XRD) with a diffractometer PANalytical, X’Pert PRO using Cu-*K*_a_ radiation (λ = 1.540 Å). Fourier-transform infrared spectroscopy (FT-IR) characterization was carried out on the BRUKER FT-IR spectrometer in the range 400–4000 cm^−1^. The porosity was investigated via nitrogen adsorption/desorption characteristics. Adsorption measurements were performed on a Micrometrics ASAP2010 volumetric adsorption apparatus.

### Electrochemical study

All electrochemical experiments were carried out at 25 °C via a conventional three-electrode cell by the use of a potentiostat (GAMRY instruments, Reference 600, Potentiostat/Galvanostate/ZRA). The utilized three electrodes were a platinum spiral wire (Pt), Ag/AgCl/KCl (sat.), and the CuNFNPs material at glassy carbon electrode (GCE) as a counter, reference electrode, and working electrode (WE), respectively. The WE was fabricated via three major steps: mechanical-polishing, electrochemical-cleaning and immobilization of the introduced CuNFNPs at the surface of GCE. Before using GCE in electrochemical experiments or modification, polishing to be like mirror smoothness should be exactly done. After the electrode polishing, it was cleaned electrochemically by the use of cyclic voltammetry (CV) measurements for 10 cycles in 0.1 mol/l of H_2_SO_4_. To fabricate the WE, a suspension of CuNFNPs in isopropanol was prepared. 10 mg of CuNFNPs was suspended in 1.5 ml of 2-propanol for 40 min. Then, a drop of 5% Nafion was added and sonicated for 20 min. A 5 µL was cast on the GCE surface and dried under room environment. The previous step was repeated 3-times until the GCE surface was completely covered. Electrochemical experiments including CV, CA and EIS were achieved by Potentiostat/Galvanostat/ZRA Gamry instrument. The electrochemical studies were investigated according to the 3-electrode system as reported before^22,23^. The measurements were repeated to test the reproducibility of the results.

## Results and Discussion

### Morphology and chemistry of the synthesized ferrite material

**Figure 1:**
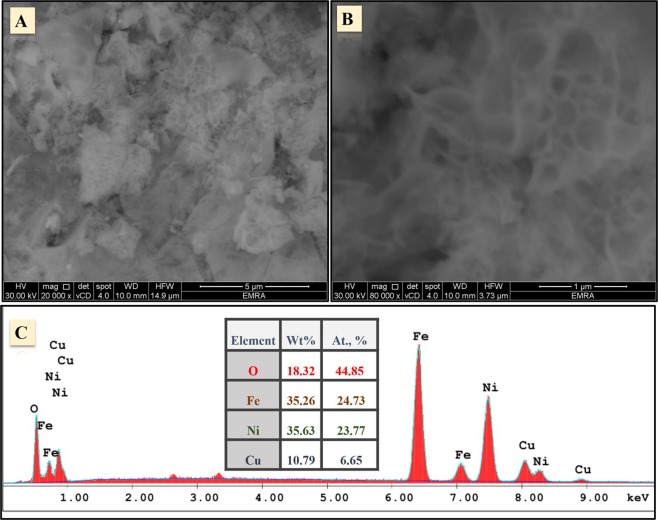
FE-SEM images of the introduced CuNFNPs material at different magnifications; 20 KX (**A**), and 80 KX (**B**), and EDX-study (**C**).

The designed copper-nickel ferrite nanoparticles (CuNFNPs) were studied by the use of FT-IR, FE-SEM, TEM, HR-TEM, and SAED to investigate the characteristics of morphology and crystallinity. Figure 1A,B shows FE-SEM images at two different-magnifications; 20 KX and 80 KX. The morphology has sponges-structure containing irregular pores. The formation of the irregular porous network can be due to the synthetic methodology which has combustion at 180 °C and followed by calcination at 400 °C. Therefore, most gases can be evaporated during these two processes and forming a porous structure. The FE-SEM-EDX of the investigated CuNFNPs was shown in Fig. 1C which displays the presence of O, Ni, Cu and Fe peaks without high-intensity peaks related to contaminants. Additionally, the absence of C-content can be used to prove the successful formation of M-oxides from M-salts. The Wt. % of the O, Ni, Cu, and Fe were found at 18.32, 35.63, 10.79 and 35.26% which indicate the formation of mixed oxides without contaminants.

**Figure 2:**
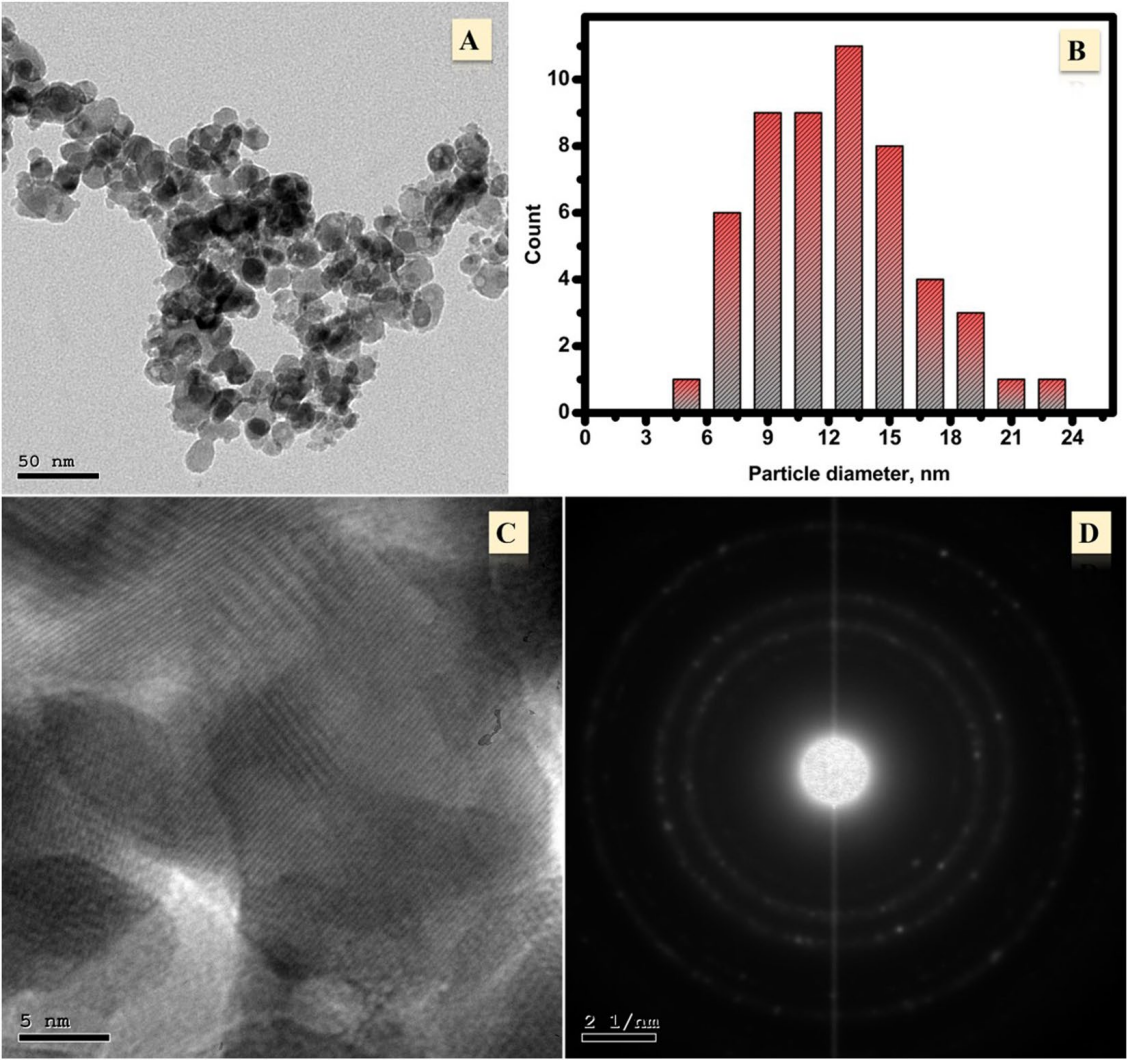
TEM analyses of the synthesized Cu-nickel ferrite including TEM-image (**A**), particle size analyses; (**B**), HR-TEM (**C**) and SAED (**D**).

TEM analyses were carried out to prove the morphological characteristics of CuNFNPs as displayed in Fig. 2. The designed material has random nano-scale and semi-spherical particles as shown in Fig. 2A. The diameter of the presented particles was found in the range of 4–24 nm and studied statistically as described in the histogram of Fig. 2B. Most of the particle’s diameter located in the range of 9–15 nm. As can be seen, 19.2% of particles have a diameter around 13 nm and 17.3% have 11 nm. Additionally, the HRTEM and SAED were carried out to study the crystallinity of the designed NPs (Fig. 2C,D, respectively). In the HR-TEM image (Fig. 2C), the metallic lattice fringes were found at 0.205 nm and 0.250 nm which related to crystal planes of (311) and (400)^24^ which can be considered as another indicator for the successful preparation of nickel ferrites cubic structure. Figure 2D displays the SAED analysis which has small bright spots as rings around a central spot having different diameters, which proves the successful synthesis of polycrystalline nickel ferrite material. To conclude, TEM analyses suggested that the utilized synthetic method can be used to prepare crystalline ferrite material as proved via FE-SEM, EDX besides TEM study.

**Figure 3:**
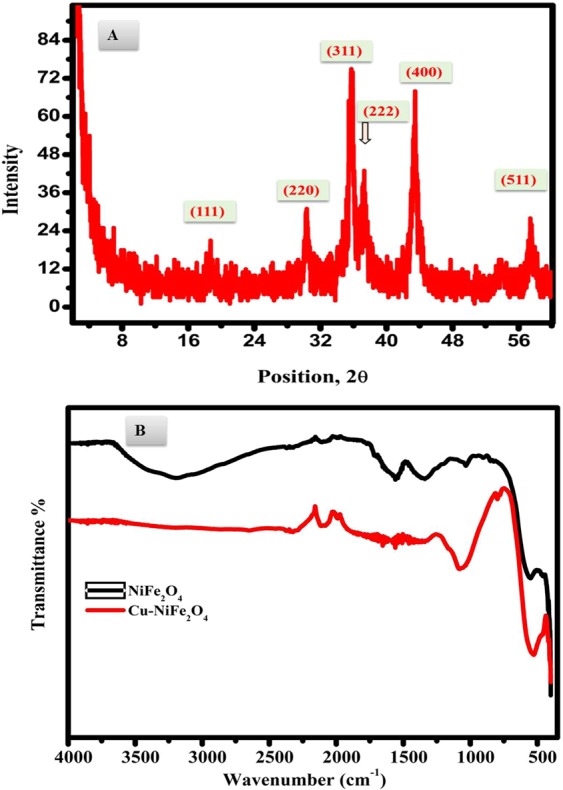
XRD-analysis of the synthesized CuNFNPs material (**A**) FT-IR spectra of nickel ferrite (black line) and Cu-doped nickel ferrite (red line) calcined at 400 °C (**B**).

XRD analysis was conducted to study the crystallinity beside the chemical content of the designed CuNFNPs (Fig. 3A). Table (1) shows the details of XRD including 2*θ*°-values, FWHM, d-spacing, relative intensities, and hkl-values. The obtained results of XRD were matching with the cubic-crystal structure of nickel ferrite (space group fd3m-227, JCPDS 00-010-0325)^17,25^. The 2*θ* peaks were located at 18.64, 30.34, 35.57, 37.39, 43.42 and 57.42°, which are corresponding to crystal planes of (111), (220), (311), (222), (400), and (511). XRD analysis indicated that no other contaminants can be seen which proved the preparation of cubic ferrite without the existence of impurities. The crystallite size was estimated using Eq. (1); Debye-Scherrer’s equation^26^, and found at 202.03 Å or 20.2 nm.

1 $$D=\frac{0.9\lambda }{\beta \,\cos \,\theta }$$

where “*λ*” is X-rays wavelength (1.541874 Å), “*β*” is the full width at half maximum (FWHM) and θ is the Bragg’s angle of the radians.

**Table 1:** XRD analyses of the designed CuNFNPs.

No.	2*θ*°	FWHM	d-spacing [Å]	Rel. Int. [%]	*hkl*
1	18.6394	1.4170	4.76054	9.20	111
2	30.3365	0.4723	2.94639	36.06	220
3	35.5707	0.4133	2.52392	100.00	311
4	43.4163	0.4723	2.08429	99.41	400
5	57.4247	0.9446	1.60474	27.17	511

In addition, the crystal parameters were calculated and found at 8.371 Å (a = b = c = 8.371 Å) which was slightly larger than that of pristine NiFe_2_O_4_ (8.339 Å). The volume of the CuNFNPs cell was estimated via the basic equation of cubic structure and found equal to 586.56 Å^3^ which was larger than that of pure nickel ferrite (579.89 Å^3^). The expansion of the crystal volume of the designed CuNFNPs can be attributed to the doping of Cu^2+^ which has slightly larger radii than Ni^2+^. The radii of Cu^2+^ and Ni^2+^ are 0.57 and 0.55 Å, respectively^27^. Therefore, the replacement of Ni^2+^ by larger size metallic ion (Cu^2+^) can increase the crystal parameters and volume as found in the designed CuNFNPs^28,29^. In conclusion, the XRD analysis proved that the prepared nanoparticles have large-size crystals of nickel-ferrite which indicates the presence of a few Cu^2+^ sites instead of Ni^2+^ sites.

FT-IR spectra of nickel ferrite and Cu-doped nickel ferrite calcined at 400 °C for 3 hrs are denoted in Fig. 3B. The spectrum reveals characteristic absorption bands: at 543 cm^−1^ and 408 cm^−1^ assigned for the Metal–Oxygen bonds^30^. These higher and lower wavenumbers are ascribed for tetrahedral and octahedral coordination sites in spinel ferrites. These bands were shifted to the lower frequency at 538 cm^−1^ and 380 cm^−1^, that is pronounced to the Fe–O and Cu, Ni–O bonds. That can be discussed due to the substitution of Ni^2+^ by metallic ion (Cu^2+^) in Cu doped ferrite sample.

**Figure 4:**
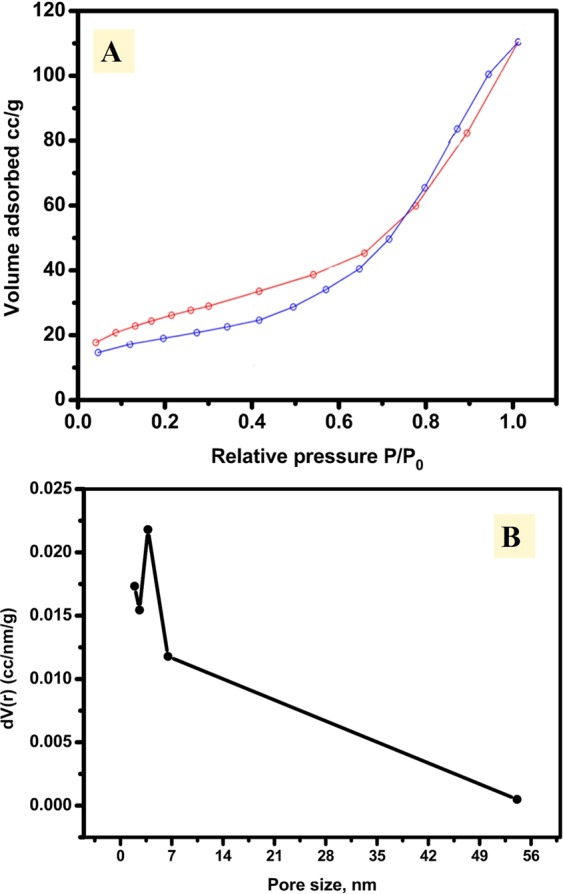
Nitrogen adsorption-desorption isotherm of the synthesized copper-nickel ferrite at 77 K (**A**) and pore-size analyses (**B**).

The surface area of the prepared CuNFNPs was investigated via nitrogen adsorption-desorption isotherm as displayed in Fig. 4A. The represented isotherm can be categorized as type IV isotherm. The adsorption and desorption processes have different characteristics and the combined behavior indicates the characteristic of mesoporous material. The desorption branch is slightly lower than the adsorption branch and it crosses over at the *P/P0* of 0.75. This behavior can be related to the slow diffusion of N_2_ molecules through the irregular shaped pores and the unreachable of thermodynamic equilibration between the branches.

The BET area had been estimated for CuNFNPs and found at 92.28 m^2^/g which is higher than the reported materials having similar chemical content^31^. The high surface area can be due to the preparation strategies which have an organic framework (citrate salt) and the calcination step after the combustion step which provides both porosity and high surface area because of the chemical decomposition and gases-evaporation. Additionally, the pore size/volume analysis was investigated as shown in Fig. 4B and the size of the pores were found between 2 and 55 nm which proves the mesoporous structure of the introduced CuNFNPs. At last, the synthesized CuNFNPs material has a porous and high surface area character which may play a role in the electrocatalysis application.

### Electrocatalytic oxidation of acetaldehyde

#### Effect of ^−^OH media

**Figure 5:**
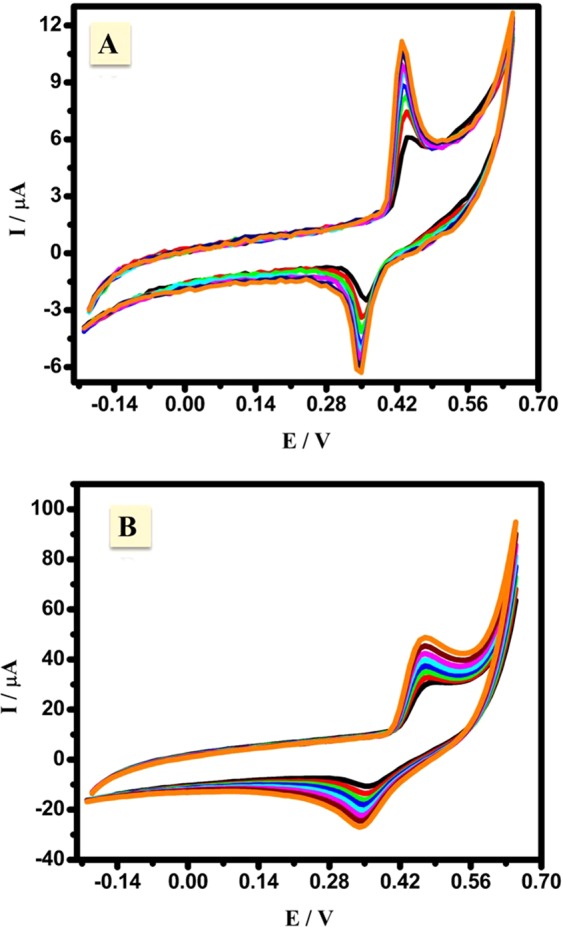
Consecutive cyclic voltammograms of the introduced CuNFNPs in ^−^OH media (0.5 M KOH) in the absence and presence of Cu-doping (NFNPs **A** and CuNFNPs **B**, respectively).

**Figure 6:**
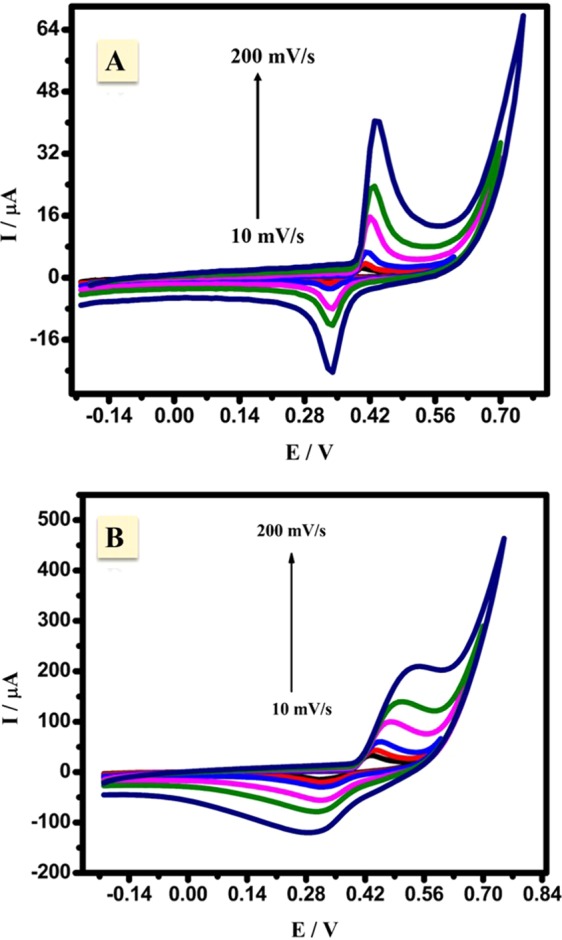
Cyclic voltammograms (CVs) of NiFe_2_O_4_ (**A**) and CuNFNPs (**B**) at different scan rates (10 to 200 mV/s) in only KOH-media.

The electrooxidation of acetaldehyde by the use of the presented CuNFNPs was studied in ^−^OH media, therefore, the effect of ^−^OH was firstly studied without acetaldehyde content. Consecutive cyclic voltammograms of the introduced CuNFNPs in -OH media (0.5 M KOH) were studied in the absence and presence of Cu-doping (NFNPs and CuNFNPs, respectively) as shown in Fig. 5A,B, respectively at 0.1 V/s. The CV behavior has redox peaks in both figures at 0.35 V and 0.43 V for the reduction and oxidation peaks, respectively. The presence of the mentioned peaks can be attributed to the Ni-sites oxidation from Ni(OH)_2_ to NiOOH. The current values were increased from 6.1 to 11.2 µA at the anodic curve and the same behavior was observed in the case of the cathodic case (from 2.5 to 6.3 µA) as the scan-number increased. After Cu-doping, the CV characteristic has high current-values compared with free-Cu one (pristine NFNPs). The enhancement of current values was also observed in both reduction and oxidation curves. Indeed, the oxidation of Ni-sites occurred in many steps and can be initiated by conversion of Ni to β-Ni(OH)_2_ and then to γ-NiOOH which accumulated at the electrode surface and increase the current values^32–34^. The effects of scan rate on the characteristics of CV profiles were studied over a range of 0.1–0.25 V/s in the presence and absence of Cu-doping as shown in Fig. 6A,B, respectively. The oxidation and reduction currents are enhanced by application higher scan rate up to 0.25 V/s, which may be due to the fast electron transfer at the interface of the electrolyte/working electrode. The enhanced current values in the presence of Cu-doping can be related to the enhancement of Ni active sites. Additionally, the improvements of both anodic and cathodic currents prove the diffusion character of the Ni-oxidation over the introduced CuNFNPs^5^.

#### Effect of acetaldehyde

**Figure 7:**
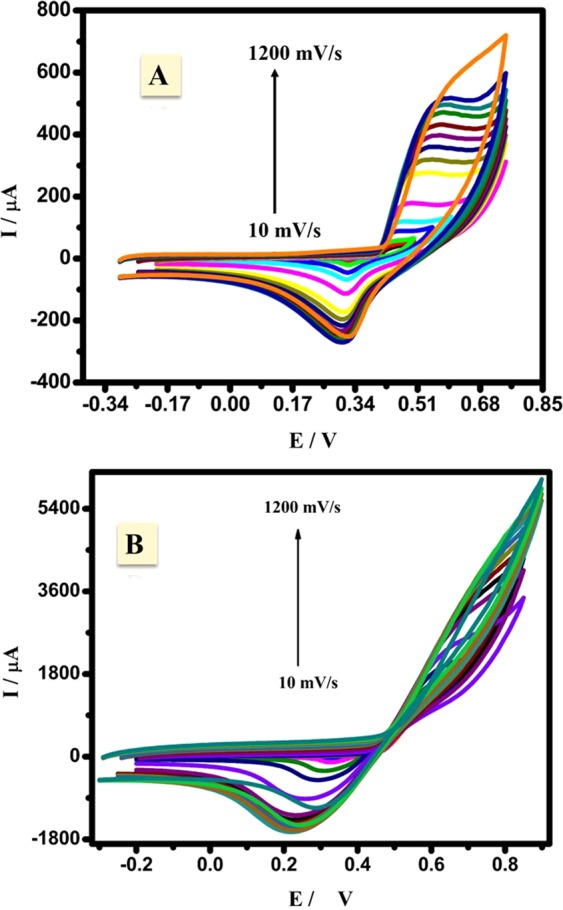
Cyclic voltammograms (CVs) of NiFe_2_O_4_ (**A**) and CuNFNPs (**B**) at different scan rates (10 to 1200 mV/s) in acetaldehyde/KOH-media.

The electrocatalytic behavior of CuNFNPs with and without Cu-doping was studied by using the acetaldehyde oxidation reaction (AOR) in alkaline medium. The AOR was investigated via cyclic voltammetry (CV) measurements at different scan rates from 10 mV/s to 1200 mV/s. The CV was firstly investigated without Cu-doping for pristine ferrite material. The anodic peak of AOR starts from 0.39 V and continue to increase from 30.1 µA to 135.8 µA at 100 mV/s. The difference between the CV in the presence and absence of acetaldehyde (Figs. 6A and 7A, respectively) for pristine NFNPs indicates the electroactivity of the ferrite material for AOR. The anodic current at the end of oxidation current is enhanced from 67.7 µA to 135.8 µA at 100 mV/s which equivalent to the double improvement in terms of the anodic current which can be considered as the main proof for study the electrooxidation reactions. The current at the end of the peak can be related to two reactions, electrooxidation and oxygen evaluation (OER). In the absence of acetaldehyde, the current is related to Ni-oxidation and OER. For acetaldehyde curves, the current is related to acetaldehyde oxidation besides OER. After Cu-doping to design CuNFNPs, The CV-curves were studied as displayed in Fig. 7B and the anodic-current curve was strongly improved if compared with the pristine NFNPs or CuNFNPs in the absence of acetaldehyde. The current at the end of the CV which includes the AOR and OER was enhanced from 135.8 µA to 1694 µA at 100 mV/s because of only Cu-doping to introduce CuNFNPs as a high-performance electrocatalyst for AOR. The role of Cu-doping in the electroactivity of CuNFNPs can be related to the structural defects of Ni, and so, higher active surface area could be provided for AOR. The presence of Cu-sites as inactive sites could greatly enhance the defects and so, increase the electron-transfer. The enhanced electroactivity after Cu-doping was found in the presence and absence of acetaldehyde as shown in (Figs. 6A and Fig. 7A) and (Figs. 6B and 7B), respectively. The CuNFNPs material showed much better current values if compared with pristine NFNPs without Cu-doping. Additionally, the observed peak potential was shifted to be higher positive as the scan rate increased which is a feature of irreversible oxidation reactions. The anodic current curve was enhanced sharply with the increase of scan rate indicating the diffusion character of the rate-determining step (RDS)^5,23,35^ which is related to the degradation of acetaldehyde during AOR. A plot of peak current (*I*_P_) values of AOR versus the square root of scan rate $(\sqrt{\upsilon )}$ at in the presence of NFNPs and CuNFNPs electrocatalysts was presented in Fig. S1A (Supporting Data). The resulting linear relationships confirmed a diffusion-controlled pathway during AOR. The variation of AOR peak potential (*E*_p_) with the log *ʋ* is depicted in Fig. S1B (Supporting Data). The irreversible performance of AOR at NFNPs and CuNFNPs electrocatalysts was established by the linear trend of these diagrams^36^. The diffusion coefficient of acetaldehyde molecules (D) at NFNPs and CuNFNPs electrocatalyst interfaces could be calculated based on the following Eqn.^37^:

2 $${I}_{p}=2.99\times {10}^{5}n{[{n}_{0}(1-\alpha )]}^{1/2}AC{D}^{1/2}\sqrt{\upsilon }$$

where *n* represents the total number of electrons involved in AOR to its products, *n*_ο_ is the number of electrons in the rate determining step, *A* is the surface area of the tested electrode, *α* is the anodic transfer coefficient and *C* is [acetaldehyde]. The following equation could be used to calculate the anodic transfer coefficient value (*α*)^38^:

3 $${E}_{p}=K+[\frac{0.03}{{n}_{0}\alpha }]\log \,\upsilon $$

where *K* is a constant. According to Eqs. 2 and 3, the D values of acetaldehyde molecules at NFNPs and CuNFNPs electrocatalysts were found to be 4.67 × 10^−7^ and 1.31 × 10^−5^ cm^2^ s^−1^, respectively. As a result, Cu-doped nickel ferrite was advantageous in enhancing the diffusion coefficient of acetaldehyde molecules at Cu_x_Ni_(1−x)_Fe_2_O_4_ by 28.05 times when compared to those at pristine NiFe_2_O_4_.

#### Material loading and chronoamperometric studies

**Figure 8:**
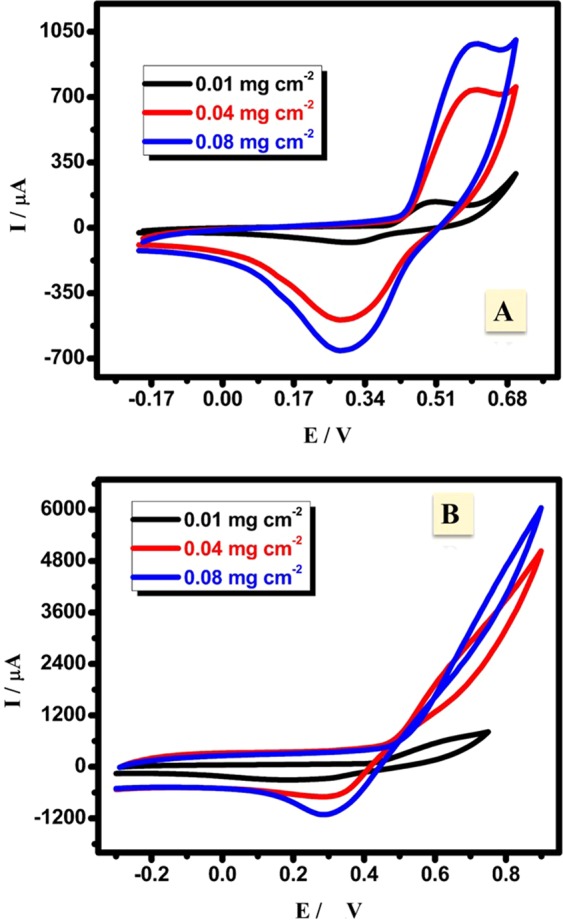
Effect of the materials loading on the CV characteristics for NiFe_2_O_4_ (**A**) and CuNFNPs (**B**).

The effect of CuNFNPs and pristine NFNPs loading on the electrocatalysis performance was investigated at 3 different loadings: 0.01, 0.04 and 0.08 g/cm^2^ (Fig. 8). The current value sharply increased when the loading changed from 0.01 to 0.04 g/cm^2^. Then, the current curve slightly enhanced after the loading increase to 0.08 g/cm^2^. These results indicate two points; the electroactivity of NFNPs and the suitable loading of 0.08 g/cm^2^. A similar characteristic of the loading effect was found in the case of CuNFNPs which proves the high-performance of the introduced CuNFNPs. Furthermore, the obtained current values in the case of CuNFNPs are much higher than that of NFNPs without Cu-doping which proves the potential of Cu-doping. In conclusion, CuNFNPs material has high electrocatalytic performance than that of NFNPs without Cu-doping and 0.08 g/cm^2^ can be utilized as the ideal loading.

**Figure 9:**
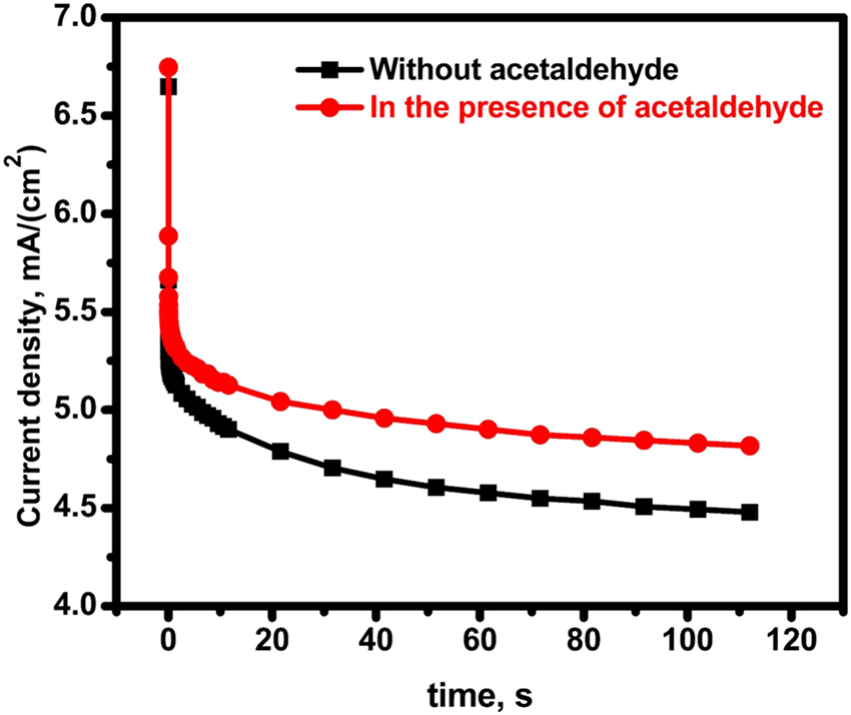
Chronoamperometric measurements of CuNFNPs in the presence and absence of acetaldehyde.

The chronoamperometric (CA) behavior of CuNFNPs material was studied in the presence and absence of acetaldehyde to study two factors; current stability and electroactivity via the difference between CA in the presence of acetaldehyde and another one without acetaldehyde as shown in Fig. 9. In the electrolyte containing acetaldehyde, the CA curve produced slightly stable, continuous and better current values with comparison to curve without acetaldehyde. The common decay at the start of the CA curve is primarily due to the rapid permeation of electroactive ions at the electrode surface. After the normal and/or expected decay, which was finished in 4.6 s, the current values remained stable up to 112 s. The value of current was changed from 5.23 to 4.82 mA/cm^2^ after the time passed from 4.6 to 112 s which means the current save around 92.2% and decayed for only less than 8%. The low decay of current values in the CA study indicates the stability of the produced current which mainly comes from the electrooxidation process. The CA outcome suggests that the introduced CuNFNPs is a promising non-precious electrocatalyst towards AOR.

#### EIS, mechanistic and comparative studies

**Figure 10:**
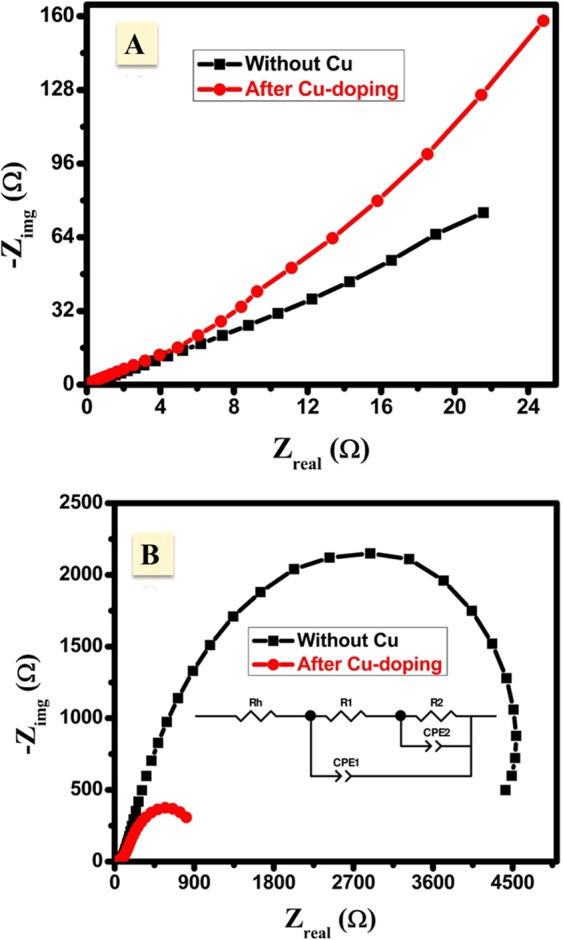
Electrochemical impedance spectroscopy (EIS) of NiFe_2_O_4_ and CuNFNPs electrodes in KOH electrolyte (**A**) and acetaldehyde/KOH-media (**B**).

To investigate the electron transfer during the AOR, EIS was carried out with and without acetaldehyde for both CuNFNPs and pristine NFNPs. Figure 10A shows the Nyquist plots of NFNPs and CuNFNPs in alkaline solution without acetaldehyde. The two investigated electrodes showed linear behavior, indicating the diffusion impedance of hydroxide-ions from the alkaline electrolyte to the working electrode surface^39^. In contrast, a semicircle was easily shown in the presence of acetaldehyde (Fig. 10B) for both NFNPs and CuNFNPs. The diameter of the seen semicircle is depending on the value of the charge transfer resistance of the designed electrodes material and the solution interface. EIS curves of CuNFNPs electrode has a much lower diameter value compared with the NFNPs without Cu-doping. The exact values of charge transfer resistance and capacitance values can be estimated via fitting the EIS-data according to the optimum equivalent circuit which can give the same data and indicate the charge transfer process. The suitable circuit was shown in the inset of Fig. 10B ^23,40^ and the estimated fitting parameters including charge transfer resistances was displayed in Table (2). In the optimum electrical equivalent, there are 3-components; Rh, CPE1/CPE2, R1/R2 which indicate the series resistance, constant phase elements and the charge-transfer resistance^41^. In AOR, there’re two processes; catalyst activation and acetaldehyde degradation. The R1/CPE1 and R2/CPE2 elements indicate the electrooxidation of Ni^2+^ as active sites and acetaldehyde, respectively. The sharp decline in the R1&R2-values after Cu-doping can be used as another proof for the successful enhancement of NFNPs via Cu-doping. The R2-value which directly indicates the acetaldehyde oxidation was decreased from 4.713 kΩ to 1.205 kΩ after Cu-doping. The enhancement of AOR after Cu-doping can be attributed to the structural defects of Ni, and so, higher active surface area could be provided for AOR. The presence of Cu-sites as inactive sites might enhance the defects and so, decrease the transfer resistance. In short, the introduced CuNFNPs material has low charge transfer impedance and high-performance towards AOR in KOH-electrolytes.

**Table 2:** Electrochemical impedance parameters as estimated from the equivalent circuit presented in Fig. 10 of the introduced CuNFNPs material.

No.	Acetaldehyde content	*R*_h_ Ω	*R*_1_ Ω	*R*_2_ kΩ	CPE1-T(+), µF	CPE1-P(+), F	CPE2-T (+), µF	CPE2-P(+), F
1	Without Acetaldehyde	71.6	885.6	4.71	48.6	0.75608	4.38E-09	4.695
Error, %		1.18	225.93	0.27	2.00	0.0081	1.12E-8	0.46
2	With Acetaldehyde	40.51	78.85	1.21	349.95	0.53248	696.14	0.80214
Error, %		0.61	7.24	0.057	88.0	0.0290	73.1	2.85

**Figure 11:**
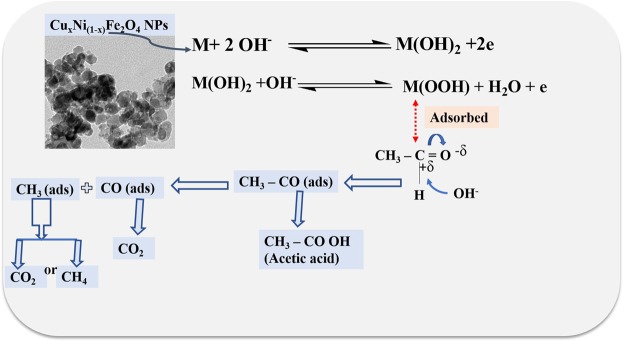
The suggested mechanism of acetaldehyde oxidation over the introduced CuNFNPs.

The mechanisms of the AOR in the alkaline medium were suggested over the surface of the CuNFNPs material as an electrocatalyst (Fig. 11). The CuNFNPs material was applied as a physical substrate and activated via CV in KOH-medium to form MOOH which has a polar bond between metal and hydroxide. Therefore, the acetaldehyde which has C=O can be adsorbed at the surface of the activated CuNFNPs. The CuNFNPs were chemically contacted to acetaldehyde and the bond between C-H in CHO group can be broken and acetaldehyde converted to CH_3_CO (adsorbed at the surface of CuNFNPs). The adsorbed CH_3_CO may be converted to acetic acid or CO_2_ or CH_4_ in the presence of OH media. The electroactivity of the CuNFNPs catalyst may be decreased due to the poisoning via the products including acetic acid and/or CO_2_. Table 3 was shown to compare the electro-activity of some reported anode materials and the introduced NFNPs and CuNFNPs. The material-performance was compared by the use of current values at the oxidation peak curves. From Table 3, it is easy to see that the synthesized CuNFNPs has a high-efficiency higher than that reported NFNPs. Additionally; the designed CuNFNPs have low electron transfer resistance and a simple preparation method.

**Table 3:** Electro-activity comparison of some reported Ni-ferrites electrocatalysts materials towards via the values of the produced current at the anodic peak.

Material	condition	Anodic Current value	References
NiFe_2_O_4_ nanoparticles synthesized by electrochemical method	100 mV/s 10 mM glucose	50 µA	^42,43^
NiFe_2_O_4_/graphene nanocomposite	250 mV/s 2 µM acetaminophen	80 µA	^44^
	250 mV/s 2 µM tramadol	60 µA	
NiFe_2_O_4_ nanoparticles integrated into carbon paste	100 mV/s 3 mM nitrite	600 µA	^45^
NiFe_2_O_4_ nanoparticles synthesized by electrochemical method	100 mV/s 10 mM NADH	14 µA	^42^
reduced graphene oxide-NiFe_2_O_4_ nanocomposites	50 mV/s 1 mM hydrazine	24 µA	^20^
NiFe_2_O_4_ synthesized via sol-gel and citric acid as a capping agent	100 mV/s 50 mM acetaldehyde	132.6 µA (1.87 mA/cm^2^)	**This Work**
	200 mV/s 50 mM acetaldehyde	312 µA (4.39 mA/cm^2^)	
Cu_x_Ni_(1−x)_Fe_2_O_4_ synthesized via sol-gel and citric acid as a capping agent	100 mV/s 50 mM acetaldehyde	1694 µA (23.86 mA/cm^2^)	
	200 mV/s 50 mM acetaldehyde	2140 µA (30.14 mA/cm^2^)	

## Conclusion

Non-precious ferrite nanoparticles were successfully fabricated via sol-gel methodology and citric acid as a capping agent. The chemistry of the introduced ferrite has Cu in addition to Ni, Fe, and O and can be symbolized as (Cu_x_Ni_(1−x)_Fe_2_O_4_) nanoparticles; (CuNFNPs). The electrocatalysis of acetaldehyde oxidation reaction (AOR) was studied by use of the introduced CuNFNPs through Cyclic-voltammetry (CV) measurements, chronoamperometric (CA) analyses and electrochemical impedance spectroscopy (EIS) to study the efficiency of the introduced ferrite as an active electrocatalyst as well as the electron transfer during AOR. The anodic-current values were sharply increased after adding acetaldehyde in the alkaline electrolytes which prove the high-efficiency of the introduced CUNFNPs for AOR. Additionally, the charge transfer resistance R2-value which directly indicates the acetaldehyde oxidation was decreased from 4.713 kΩ to 1.205 kΩ after Cu-doping. The enhanced electroactivity after Cu-doping was found in the absence and the presence of acetaldehyde. The Cu-role can be concluded in the enhancement of Ni-structural defects and active sites and so, increase the generated current, electrical conductivity and electron-transfer. In short, the CV, CA and EIS studies showed the high oxidation current and low charge transfer resistance can be produced via CuNFNPs. This study introduces a non-precious Cu-doped-Ni-ferrite as an electrocatalyst towards acetaldehyde electrooxidation in alkaline media.

## Supplementary information

Supplementary Information.

## Data Availability

The authors confirm that the data supporting the findings of this study are available within the article and its supplementary materials.
