# Supplementary material for: A facile chemical synthesis of CuxNi(1−x)Fe2O4 nanoparticles as a nonprecious ferrite material for electrocatalytic oxidation of acetaldehyde
Source: Sci Rep. 2020 Feb 17;10:2761. doi: 10.1038/s41598-020-59655-3 (PMC7026399; doi:10.1038/s41598-020-59655-3)
Supplement: Supplementary file 1 — Supplementary Information. [file 41598_2020_59655_MOESM1_ESM.docx]

**A facile chemical synthesis of Cu_x_Ni_(1-x)_Fe_2_O_4_ nanoparticles as a nonprecious ferrite material for electrocatalytic oxidation of acetaldehyde**

Mai M. Khalaf ^a,b,^ , Hany M. Abd El-Lateef ^*a,b^, Ahmed O. Alnajjar^a^, Ibrahim M A Mohamed ^b,**^

*^a^* Department of Chemistry, College of Science, King Faisal University, P.O. Box 380 Al Hofuf 31982 Al-Ahsa, Saudi Arabia

*^b^ Department of Chemistry, Faculty of Science, Sohag university, Sohag 82524, Egypt*

*** Corresponding authors: Email: * [**hmahmed@kfu.edu.sa**](mailto:hmahmed@kfu.edu.sa)**,** [**hany_shubra@yahoo.co.uk**](mailto:hany_shubra@yahoo.co.uk) (H.M. Abd El-Lateef)

** **ibrahim_mohamed@science.sohag.edu.eg** (I M A Mohamed).

**Figure S1**: (A) Variation of *I*_p_ of AOR with the value of scan rate. (B) Plot of peak potential value against the logarithm of scan rate.
